# Editorial: The fiber profile of skeletal muscles as a fingerprint of muscle quality

**DOI:** 10.3389/fphys.2022.1105252

**Published:** 2022-12-09

**Authors:** Emiliana Giacomello, Luana Toniolo

**Affiliations:** ^1^ Department of Medicine, Surgery and Health Sciences, University of Trieste, Trieste, Italy; ^2^ Laboratory of Muscle Biophysics, Department of Biomedical Sciences, University of Padova, Padova, Italy

**Keywords:** skeletal muscle, fiber size, myosin heavy chain, oxidative capacity, metabolic activity, fiber profile

Skeletal muscles are organs with a unique plasticity, which respond to the rapid changes of the organism activity, nutritional habits, health conditions, genetic background, and other factors (Schiaffino and Reggiani, 2011; Sirago et al., 2022). Traditionally, the ability to adapt to physiological changes is ascribed to the evidence that a muscle is composed by multiple fibers that have different contractile characteristics. Actually, skeletal muscle fibers differ one from another in the contractile apparatus composition, calcium handling properties, number of mitochondria, capillarization, intramyocellular lipids, glycogen content, and morphological features (Schiaffino and Reggiani, 2011; Giacomello et al., 2020). In general, the isoforms of Myosin Heavy Chain (MHC) expressed in a muscle fiber, are considered the gold standard in the correlation of the morphological, contractile, and metabolic properties (Burke et al., 1971; Schiaffino and Reggiani, 2011). Besides, numerous other proteins, such as Troponins (Rasmussen and Jin) adapt their expression to meet the contractile properties of a determined fiber (Schiaffino and Reggiani, 2011).

Here, we refer to the term “fiber profile” to all the features, such as MHC expression, morphology and metabolic activity, that characterize a single muscle fiber. Usually, slow contracting fibers, have a smaller cross-sectional area (CSA), are rich in mitochondria, have a low content of glycogen, and are surrounded by numerous capillaries. Vice versa, fast contracting fibers display a wider CSA, have low mitochondrial activity, high glycogen content, and less capillaries.

Skeletal muscle adaptation to different physiological conditions involves the modification of these characteristics, entailing MHCs transition, alteration of metabolic capacity and morphological features. Although the MHC isoform/s expressed have been considered to determine the metabolic properties of a muscle fiber, recent data report some exceptions.

In this context, the current issue has been motivated by evidences showing that both in rodent and human muscles, morphological and metabolic characteristics of fibers expressing the same MHCs can significantly diverge.

In 2012, Bloemberg and Quadrilatero (Bloemberg and Quadrilatero, 2012) in a systematic immunofluorescence analysis of MHCs, found that the type 2A muscle fibers were significantly smaller in extensor digitorum longus compared to soleus muscle, in both rats and mice. This topic was further approached and investigated in human muscles by Ortenblad and collaborators (Ørtenblad et al., 2018), who showed that, equally trained leg and arm muscles from cross-country skiers, have a different percentage of type 1 and 2A fibers. And, interestingly, arm muscles present wider type 2A fibers, with higher mitochondrial content, and more capillarized compared to leg muscles. This behavior has been suggested to respond the need of producing great force and power in short periods.

Interestingly, data from our laboratory suggest that there is a modulation of the metabolic capacity also during age. In fact, the analysis of the fiber profile of the fast contracting muscle tibialis anterior in C56BL/6J mice, reveals that type 2A and 2X, but not 2B, fibers become more positive to succinate dehydrogenase staining with age, indicating a specific increase of mitochondrial activity in these fibers (Giacomello et al., 2020).

The modifications to the metabolic activity, as shown by Ortenblad and collaborators in their further research on leg and arm muscles of cross-country skiers (Gejl et al.), have a functional consequence. In fact, the single fiber analysis of type 1 fibers, showed that they display a better specific force, and an increased calcium sensivity in leg compared to arm muscles. The divergent properties of the same type fibers in lower an upper limbs muscles, can also explain the different susceptibility to sarcopenia of legs and arms (Venturelli et al., 2015).

The evidence that the metabolic properties of a skeletal muscle cell can change independently from the myosin isoform expressed, provide to muscle fibers a further level of flexibility, and suggest to refer to the term “muscle plasticity” in a more ample view.

This concept can have also an important implication in the search of strategies to improve muscle health, and can partially explain the high variability of data found in the literature. In this context, the systematic review from Wang and collaborators (Wang et al.), which reports the effects of chronic exercise on autophagy-related proteins in aging muscle, suggest as limitation of the study not only the different exercise protocol, but also the variability of samples, the specific muscle analyzed, and type of model used.

Moreover, it should be kept in account that although muscle fibers are essential actors, muscle contraction is possible thanks their strict communication with nerves, connective tissue, and capillaries. For example, ageing and other pathological conditions entail capillary rarefication, which in turn creates a hypoxic condition that can be detrimental for muscle fibers. Muscle stretch (Hotta and Muller-Delp), the use of antioxidants (Toniolo et al., 2021), the application of dietary regimens, or other pharmacological interventions can improve muscle health by modulating the capillary network.

The knowledge of muscle composition could be of fundamental importance also in various clinical aspects as highlighted by the report of Yamashita and collaborators (Yamashita et al.), who delve into the muscle dependent response to myorelaxants. Based on the past evidence that myorelaxants have different potencies depending on the fiber composition of distinct muscles, in their report, Authors suggest that the different response to myorelaxants in fast and slow fibers, depends on the Acetylcholine receptors subunits expressed at the level of the neuromuscular junction.

In summary, the present Research Topic collects a research article (Gejl et al.), a brief research report (Yamashita et al.), two reviews (Rasmussen and Jin; Wang et al.) and a mini review (Hotta and Muller-Delp), which implement previous knowledge, and provide new information on skeletal muscle fiber profiles in distinct physiological conditions. In a future perspective, such an approach could help to create of a sort of fingerprint of different muscles in different conditions, allowing to understand the heterogeneity of muscles’ response to distinct physiological needs. Moreover, in the growing field of the precision medicine, the prediction of fiber-specific parameters that assure a good quality muscle, could be essential to promote health strategies.
